# Cumulative live birth rate in mild versus conventional stimulation in progestin-primed ovarian stimulation protocols for individuals with low prognosis

**DOI:** 10.3389/fendo.2023.1249625

**Published:** 2023-11-14

**Authors:** Junwei Zhang, Mingze Du, Caihua Zhang, Yanli Wu, Yichun Guan

**Affiliations:** ^1^ The Reproductive Center, The Third Affiliated Hospital of Zhengzhou University, Zhengzhou, Henan, China; ^2^ Obstetrics and Gynecology, The Third Affiliated Hospital of Zhengzhou University, Zhengzhou, Henan, China

**Keywords:** mild stimulation, conventional stimulation, cumulative live birth rate, low prognosis, progestin-primed ovarian stimulation protocol

## Abstract

**Objective:**

The purpose of this study was to evaluate the cumulative live birth rate (CLBR) of mild stimulation and conventional stimulation for the low-prognosis population undergoing PPOS protocols.

**Methods:**

This was a retrospective cohort study. We included women with a low prognosis. All women underwent PPOS protocols, and the starting gonadotropin (Gn) dose was 150 IU or 300 IU. The primary outcome measure was CLBR. The secondary outcome measures were the number of oocytes retrieved, number of 2PN oocytes and number of available embryos.

**Results:**

In total, 171 women with mild stimulation and 1810 women with conventional stimulation met the criteria. In the PSM model, 171 mild stimulation cycles were matched with 513 conventional stimulation cycles. The gonadotropin dosage in the mild stimulation group was significantly lower than that in the conventional stimulation group (1878.6 ± 1065.7 vs. 2854.7 ± 821.0, P<0.001). The numbers of oocytes retrieved, 2PN oocytes, available embryos and high-quality embryos were also higher in the conventional stimulation group than in the mild stimulation group (P<0.05). There was no significant between-group difference in the cumulative clinical pregnancy rate (26.3% vs. 27.5%, P=0.77). The CLBR after mild stimulation was similar to that after conventional stimulation (21.1% vs. 22.0%, P=0.79).

**Conclusion:**

In our study, we found that the CLBRs of mild stimulation and conventional stimulation were similar, despite conventional stimulation resulting in significantly more oocytes and embryos. Thus, mild stimulation can be considered an option for women with a low prognosis in PPOS protocols.

## Background

The introduction of ovarian stimulation (OS) is a notable breakthrough in assisted reproductive technology (ART). OS is used to collect multiple oocytes and generate multiple embryos that can be available for transfer, thus increasing the efficacy of ART (1). The traditional OS approach is to use GnRH analogs combined with exogenous gonadotropins (Gns) to inhibit the endogenous LH peak, thereby achieving the purpose of inhibiting ovulation and developing multiple follicles (2). There has not been a major update to the OS protocols until recently, when progestin-primed ovarian stimulation (PPOS) protocols were proposed (3). The PPOS protocols utilize the anti-positive feedback effect of progesterone, and multiple studies have demonstrated its clinical efficacy and safety (3–5), especially in women with a low prognosis (6).

Patients with a low prognosis are characterized mainly by their decreased ovarian function, and the clinical diagnosis is based mainly on the Poseidon criteria (7) or the Bologna consensus (8). Altogether, the Poseidon criteria comprehensively consider ovarian biomarkers, the number of oocytes retrieved, the age-related embryo aneuploidy rate and ovarian sensitivity to exogenous Gn, so it has been widely used in recent years (9, 10); however, treatment of the low-prognosis population remains challenging, given the few oocytes retrieved and low number of available embryos in this group (11).

The intensity of OS in this population has been a matter of debate over the last decade (1, 12). On the one hand, given the previous study, the small benefit of oocyte retrieval or fresh-cycle live births with high doses of Gn in low-prognosis patients remains controversial in terms of Gn dosage (1, 13, 14). In particular, there is debate regarding whether mild stimulation is comparable to conventional stimulation for low-prognosis populations, with studies yielding conflicting results (1). On the other hand, there are currently no efficacy analyses of Gn doses in PPOS protocols. Moreover, the main observation indicators of the current studies are limited mainly to the number of oocytes retrieved or the live birth rate (LBR) of fresh embryo transfer cycles. Nevertheless, it is more than evident that the cumulative live birth rate (CLBR), which includes all subsequent frozen-thawed embryo transfer (FET) outcomes, is a more comprehensive and meaningful evaluation measure for assessing the effectiveness of an ART cycle and provides more useful information to patients (15). Therefore, the purpose of this study was to evaluate the CLBR of mild stimulation and conventional stimulation for a low-prognosis population undergoing PPOS protocols.

## Methods

### Population

This was a retrospective cohort study. This study was approved by the review board of the Third Affiliated Hospital of Zhengzhou University (2022–050–01). The study involved women who underwent their first IVF/ICSI cycles at the Reproductive Center of Third Affiliated Hospital of Zhengzhou University between January 2017 and January 2020. We included women with a low prognosis diagnosed based on the Poseidon criteria. Only Poseidon 3 or 4 groups were included for analysis, and the specific criteria were as follows: females aged <35 years with ovarian biomarker analysis showing AFC<5 and/or AMH<1.2 ng/ml were classified as Poseidon group 3, and females aged ≥35 years with ovarian biomarker analysis showing AFC<5 and/or AMH<1.2 ng/ml were classified as Poseidon group 4[5]. All women underwent PPOS protocols, and the starting gonadotropin (Gn) dose was 150 IU or 300 IU, each patient was included only once. We excluded women with adenomyosis, hydrosalpinx suggested by vaginal ultrasound, uterine malformations, endometrial polyps, preimplantation genetic testing (PGT), donor oocytes or cycle records that were missing important data.

### Progestin-primed ovarian stimulation protocol and grouping

The details regarding the implementation of the PPOS protocols have been described in our previous study (16). OS was initiated on the second or third day of the menstrual cycle. Patients were administered 6 mg of medroxyprogesterone acetate (MPA) (Beijing Zhong Xin Pharmaceutical, China) combined with human menopausal gonadotropin (hMG) (Anhui Fengyuan Pharmaceutical, China) at a dose of 150 or 300 IU/day. For mild stimulation, ovulation was initiated with 150 IU hMG. For conventional stimulation, ovulation was initiated with 300 IU hMG. Follicle growth was monitored by vaginal ultrasound combined with serum hormone analysis. Triptorelin (100 μg) (Ferring International Center SA, Germany) and 2000 IU of human chorionic gonadotropin (hCG) (Lizhu Pharmaceutical Trading, China) were administered to induce oocyte maturation when the diameter of the dominant follicle was greater than 20 mm or when at least three follicles reached 18 mm. Oocyte retrieval was performed 36 hours later. Fertilization was carried out *in vitro* by IVF or ICSI, depending on the semen parameters.

### Embryo transfer and endometrial preparation protocols

For PPOS protocols, due to the effect of exogenously applied progesterone on the endometrium, whole embryos were frozen and, subsequently, FET was performed. Endometrial preparation for FET was performed by means of the natural cycle for women with regular menstrual cycles and spontaneous ovulation; the artificial/induced ovulation cycle was used for women with irregular menstrual cycles; and downregulation + the artificial cycle was used for women with endometriosis. Follicle and endometrial scanning was performed by vaginal ultrasound, and embryo or blastocyst transfer was performed using abdominal ultrasound after 3 or 5 days of endometrial development with progesterone. Routine corpus luteum support, namely, oral dydrogesterone (2 times daily, 10 mg once) (Abbott Co. America) and intravaginal administration of 90 mg of a progesterone sustained-release vaginal gel (Merck Co. Germany), was given. Corpus luteum support was continued until at least 55 days after transfer if pregnancy occurred.

### Outcome measures and definition

The primary outcome measure was CLBR, defined as at least one live birth resulting from one aspirated ART cycle, including all FET cycles, until one delivery with a live birth occurred or until all embryos were used, whichever occurred first. The delivery of a singleton, twin, or other multiple was registered as one delivery. We took a conservative approach to assume the CLBR, which means that couples who discontinued treatment would have zero change in conceiving. The observation and follow-up time was 2 years.

The secondary outcome measures were the number of oocytes retrieved, number of 2PN oocytes and number of available embryos.

### Statistical analysis

All statistical management and analyses were performed using SPSS software, version 22.0.

A prospective score matching (PSM) model was applied to balance baseline characteristics, including maternal age, BMI, duration of infertility, type of infertility, infertility diagnosis, basal serum FSH level, AMH and antral follicle count (AFC), between the mild stimulation group and conventional stimulation group. The propensity score was obtained from a logistic regression model. Patients with mild stimulation were matched with patients with conventional stimulation at a 1:3 ratio based on the propensity score with a standard caliper width of 0.2.

Continuous variables are expressed as the mean ± SD, and Student’s t-test was used to assess between-group differences properly. Categorical variables are represented as the number of cases (n) and percentage (%). The means from chi-square analyses were used to assess the differences between groups, with Fisher’s exact test used when necessary.

All patient data and follow-up information were obtained from the electronic medical record system of the reproductive center of the Third Affiliated Hospital of Zhengzhou University. A two-sided P value <0.05 was considered statistically significant.

## Results

### Study population

**Figure 1:**
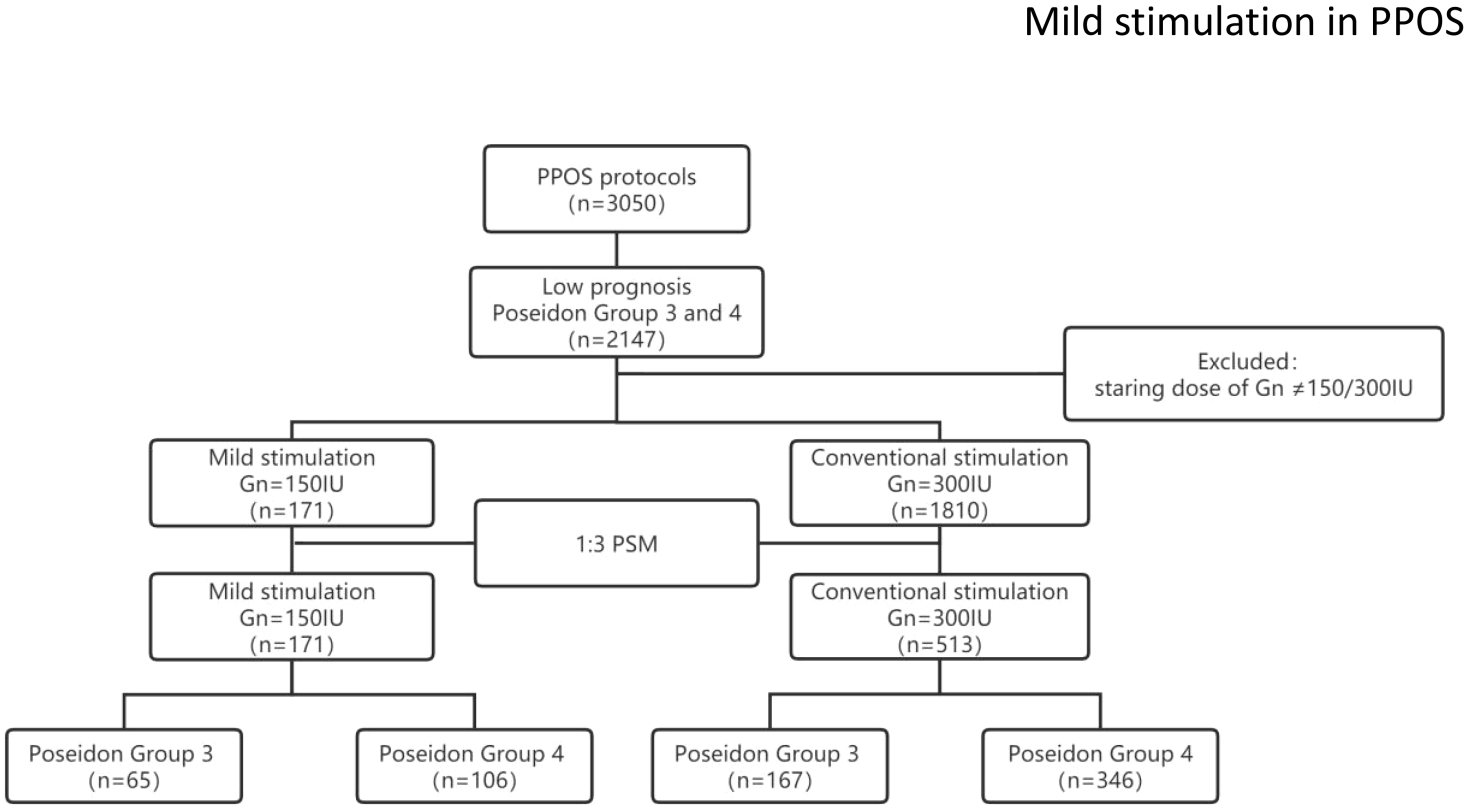
The flow chart of the study population.

A total of 171 women with mild stimulation and 1810 women with conventional stimulation met the Poseidon criteria for Poseidon group 3 or 4. After balancing the baseline characteristics using the PSM model, 171 mild stimulation cycles were matched with 513 conventional stimulation cycles. The study flow chart is shown in **Figure 1**.

### Baseline characteristics

After postmatching analysis, the baseline characteristics, including maternal age, paternal age, BMI, duration of infertility, type of infertility, indication for IVF/ICSI, basal serum FSH level, AMH and AFC, were comparable between the mild stimulation and conventional stimulation groups. The detailed comparison of the groups is shown in **Table 1**.

**Table 1:** Baseline characteristics of low-prognosis women between the two groups.

	150 IU	300 IU	P value
	(n =171)	(n =513)	
Maternal age(year)	37.4 ± 6.4	37.6 ± 5.7	0.74
Paternal age(year)	38.0 ± 7.4	37.9 ± 6.4	0.82
Body mass index (kg/m2)	24.3 ± 3.1	24.3 ± 3.1	0.98
Duration of Infertility (year)	4.1 ± 4.3	4.4 ± 4.1	0.33
Type of infertility			0.63
Primary infertility	31.0(53/171)	29.0(149/513)	
Secondary infertility	69.0(118/171)	71.0(364/513)	
Indication of IVF/ICSI			0.71
Tubal factor	14.0(24/171)	13.6(70/513)	
Male factor	2.3(4/171)	3.7(19/513)	
Low-prognosis	29.2(50/171)	25.9(133/513)	
Others	54.4(93/171)	56.7(291/513)	
Basal serum FSH level(IU/L)	10.8 ± 5.7	10.9 ± 5.5	0.77
AMH(ng/ml)	1.4 ± 2.5	1.4 ± 1.5	0.67
Basal antral follicle count	4.4 ± 2.9	4.4 ± 2.4	0.99

Data are presented as mean ± SD for continuous variable and n (%) for categorical variable.

### Clinical and pregnancy outcomes

The Gn dosage in the mild stimulation group was significantly lower than that in the conventional stimulation group (1878.6 ± 1065.7 vs. 2854.7 ± 821.0, P<0.001). On the trigger day, serum LH and E2 were higher in the conventional stimulation group (P<0.001). The number of follicles ≥14 mm, ≥16 mm or ≥18 mm was higher in the conventional stimulation group (P<0.001). The numbers of oocytes retrieved, 2PN oocytes, available embryos and high-quality embryos were also higher in the conventional stimulation group than in the mild stimulation group (P<0.05). The frequency of the cumulative clinical pregnancy rate after mild stimulation was similar to that after conventional stimulation (26.3% vs. 27.5%, P=0.77). There was no significant between-group difference in the CLBR (21.1% vs. 22.0%, P=0.79) (**Table 2**).

**Table 2:** Clinical and pregnancy outcomes of low-prognosis women between the two groups.

	150 IU	300 IU	P value
	(n =171)	(n =513)	
Dosage of gonadotropins (IU)	1878.6 ± 1065.7	2854.7 ± 821.0	<0.001
LH values on the trigger day(mIU/ml)	6.1 ± 5.0	4.6 ± 3.4	<0.001
E2 values on the trigger day(pg/ml)	807.7 ± 543.4	1153.4 ± 683.2	<0.001
Endometrial thickness on the trigger day(mIU/ml)	7.4 ± 1.8	7.5 ± 1.8	0.86
Fertilization method			0.72
IVF	84.8(145/171)	83.6(429/513)	
ICSI	15.2(26/171)	16.4(84/513)	
No. of follicles≥14 mm on the trigger day	2.2 ± 1.4	3.2 ± 1.7	<0.001
No. of follicles≥16 mm on the trigger day	1.8 ± 1.1	2.5 ± 1.4	<0.001
No. of follicles≥18 mm on the trigger day	1.1 ± 0.9	1.6 ± 1.1	<0.001
No. of oocytes retrieved	2.3 ± 1.8	3.2 ± 2.0	<0.001
No. of 2PN	1.5 ± 1.3	2.1 ± 1.6	<0.001
No. of available embryos	1.2 ± 1.2	1.7 ± 1.5	<0.001
No. of high-quality embryos	0.8 ± 1.0	1.0 ± 1.2	0.02
Cumulative clinical pregnancy rate (%)	26.3(45/171)	27.5(141/513)	0.77
Cumulative live birth rate (%)	21.1(36/171)	22.0(113/513)	0.79

Data are presented as mean ± SD for continuous variable and n (%) for categorical variable.

The cumulative clinical pregnancy rate and CLBR were stratified according to the Poseidon criteria, namely, Poseidon 3 and Poseidon 4, as specifically described in **Table 3**. There were no statistically significant differences in cumulative clinical pregnancy rates and CLBR between the mild stimulation and conventional stimulation groups.

**Table 3:** Pregnancy outcomes of patients in Poseidon group 3 and group.

	POSEIDON 3			POSEIDON 4		
	150 IU (n=65)	300 IU (n=167)	P value	150 IU (n=106)	300 IU (n=346)	P value
Cumulative clinical pregnancy rate (%)	41.5(27/65)	43.7(73/167)	0.76	17.0(18/106)	19.7(68/346)	0.54
Cumulative live birth rate (%)	35.4(23/65)	38.3(64/167)	0.68	12.3(13/106)	14.2(49/346)	0.62

Data are presented as n (%) for categorical variable.

## Discussion

According to our findings, while conventional stimulation results in more oocytes retrieved and more available embryos than mild stimulation, the cumulative clinical pregnancy rate and CLBR do not significantly differ between the two stimulation strategies.

Although several previous studies have been published on this topic, none of them analyzed Gn dosage for PPOS protocols, and the vast majority have focused on the number of embryos or LBR of fresh embryo transfer, lacking the analysis of CLBR. To the best of our knowledge, only two trials have analyzed the CLBR of poor ovarian response (POR) patients treated with different Gn dosages (17, 18). One was a multicenter prospective cohort study that analyzed AFC-based individualized FSH dosing or standard FSH dosing (FSH=150 IU). The CLBR in the mild stimulation group was 13.3% (16/120), which is comparable to that of conventional stimulation, at 9.02 (12/113) (RR (95%)=1.26 (0.62-2.54)). Conventional stimulation was more expensive (delta costs/woman = €275 (95% CI, 40 to 499)) (17). Another was a single-center prospective randomized controlled trial, including 191 patients who met the Bologna criteria of POR (97 with mild stimulation and 94 with conventional stimulation). A higher number of retrieved oocytes (P = 0.003) and embryos (P = 0.029) were obtained in the conventional stimulation, while the CLBR (OR 1.103; 95% CI 0.53 to 2.28; P = 0.791) was comparable between the two groups. A meta-analysis of these two studies also suggested that there was no significant difference in CLBR between the two protocols (RR=1.15; 95% CI: 0.73-1.81) (1). This is consistent with our findings as a whole; mild stimulation resulted in a significantly lower oocyte yield and fewer embryos but did not affect the CLBR. Although this was a retrospective cohort study, the number of cycles was larger than that in previous studies, and after PSM, the basic data of the patients were comparable.

The comparison of the number of oocytes retrieved between the two protocols is controversial. The meta-analysis included 13 studies; 2516 women showed a significantly lower oocyte yield with mild stimulation than with conventional stimulation (MD=- 0.80; 95% CI: -1.28 to -0.32), but the certainty was very low (1). Inconsistent with this result, Abdel Mohsen et al. showed a comparable number of retrieved oocytes. Such conflict may result from broader inclusion criteria and smaller sample sizes, with 60 women with one or more previous failed IVF cycles being recruited (19).

For the OS strategy, the PPOS protocol was first reported by Kuang et al. in 2015 (3). The theoretical basis of PPOS protocols is the anti-positive feedback effect of progesterone and the multiple follicular wave patterns of human follicle recruitment, which have been confirmed in previous studies (20–22). This protocol can not only effectively suppress the early-onset LH surge and reduce the cycle cancellation rate but also obtain satisfactory clinical outcomes and safety (4, 6, 23). Although this protocol will reduce endometrial receptivity due to the influence of progesterone and cannot perform fresh embryo transfer, with improvements in vitrification and FET technology, the pregnancy rate for FET cycles has been significantly improved. In recent years, PPOS has quickly become a new and widely used OS protocol due to its effectiveness, safety, simple operation, oral administration and economical aspects (4, 6, 23). However, due to the short clinical application time of the PPOS regimen, to the best of our knowledge, there is currently no relevant research on the intensity of Gn stimulation. This study is the first analysis of the application of mild stimulation and conventional stimulation in the PPOS protocol.

Based on our results, conventional stimulation resulted in a higher number of oocytes retired and a higher number of embryos, but the CLBR was comparable to that of mild stimulation. The mechanistic basis for this observation is not yet clear, but the main assumptions are as follows. On the one hand, it could be assumed that for patients with low prognosis, it is difficult to improve the final clinical outcome, even with more intense stimulation. Thus, adopting mild stimulation and reducing Gn consumption and economic cost would be the main factors to consider. On the other hand, it is hypothesized that high Gn doses may have a negative impact on oocyte or embryo quality (12, 24), but this assumption is controversial. One study suggested that euploidy rates and LBRs after the transfer of euploid embryos are not significantly influenced by Gn dosage, duration of OS or estradiol level (25).

To the best of our knowledge, this study is the first to analyze the starting dose of Gn in the PPOS protocol. Considering the relatively short clinical application time of the PPOS protocol, it is clinically meaningful to analyze its different starting doses to optimize the clinical protocol. A significant strength of this study is its inclusion of patients with a low ovarian prognosis in accordance with Poseidon groups 3 and 4 and its use of the CLBR as the observation parameter rather than a single cycle pregnancy rate or LBR. Few studies have focused on “CLBR”, the most comprehensive measure of success in IVF, as the outcome. However, this study also has certain limitations. This was a retrospective cohort study and was affected by interference from confounding factors. However, to reduce the influence of important confounders, this study used a PSM model to balance the baseline characteristics between mild stimulation and conventional stimulation.

## Conclusions

Based on our study, we found that the CLBR of mild stimulation was similar to that of conventional stimulation, despite conventional stimulation resulting in significantly more oocytes and embryos. In conclusion, mild stimulation can be considered as an option for women with a low prognosis undergoing PPOS protocols.

## Data availability statement

The raw data supporting the conclusions of this article will be made available by the authors, without undue reservation.

## Ethics statement

This study was approved by the ethics committee of The Third Affiliated Hospital of Zhengzhou University (2022–050–01). The studies were conducted in accordance with the local legislation and institutional requirements. Written informed consent for participation was not required from the participants or the participants’ legal guardians/next of kin because this was a retrospective cohort study.

## Author contributions

JZ and YG designed the study and selected the population to be included and excluded. JZ and MD were involved in the data extraction and analysis. CZ and YW reviewed the data. JZ was involved in drafting this article. All authors contributed to the article and approved the submitted version.
